# Hormetic-Like Effects of L-Homocysteine on Synaptic Structure, Function, and Aβ Aggregation

**DOI:** 10.3390/ph13020024

**Published:** 2020-02-02

**Authors:** Carla Montecinos-Oliva, Macarena S. Arrázola, Claudia Jara, Cheril Tapia-Rojas, Nibaldo C. Inestrosa

**Affiliations:** 1 Centro de Envejecimiento y Regeneración (CARE); Departamento de Biología Celular y Molecular; Facultad de Ciencias Biológicas, Pontificia Universidad Católica de Chile, Santiago 8331150, Chile; cjmontec@uc.cl (C.M.-O.); arrazola.ms@gmail.com (M.S.A.); 2 Center for Integrative Biology, Faculty of Sciences, Universidad Mayor de Chile, Santiago 8580745, Chile; 3 Laboratory of Neurobiology of Aging, Centro de Biología Celular y Biomedicina (CEBICEM), Facultad de Medicina y Ciencia, Universidad San Sebastián, Santiago 7510156, Chile; cjcjarao@gmail.com; 4 Centro de Excelencia en Biomedicina de Magallanes (CEBIMA), Universidad de Magallanes, Punta Arenas 6213515, Chile

**Keywords:** Aβ oligomers, Alzheimer’s disease, excitotoxicity, hyperhomocysteinemia, homocysteine, hormesis, methionine, neurodegenerative diseases, oxidative stress

## Abstract

Alzheimer’s Disease (AD) is the primary cause of dementia among the elderly population. Elevated plasma levels of homocysteine (HCy), an amino acid derived from methionine metabolism, are considered a risk factor and biomarker of AD and other types of dementia. An increase in HCy is mostly a consequence of high methionine and/or low vitamin B intake in the diet. Here, we studied the effects of physiological and pathophysiological HCy concentrations on oxidative stress, synaptic protein levels, and synaptic activity in mice hippocampal slices. We also studied the in vitro effects of HCy on the aggregation kinetics of Aβ_40_. We found that physiological cerebrospinal concentrations of HCy (0.5 µM) induce an increase in synaptic proteins, whereas higher doses of HCy (30–100 µM) decrease their levels, thereby increasing oxidative stress and causing excitatory transmission hyperactivity, which are all considered to be neurotoxic effects. We also observed that normal cerebrospinal concentrations of HCy slow the aggregation kinetic of Aβ_40_, whereas high concentrations accelerate its aggregation. Finally, we studied the effects of HCy and HCy + Aβ_42_ over long-term potentiation. Altogether, by studying an ample range of effects under different HCy concentrations, we report, for the first time, that HCy can exert beneficial or toxic effects over neurons, evidencing a hormetic-like effect. Therefore, we further encourage the use of HCy as a biomarker and modifiable risk factor with therapeutic use against AD and other types of dementia.

## 1. Introduction

Homocysteine (HCy) is a sulfur-containing amino acid and a byproduct of methyl-transfer reactions due to methionine metabolism [1]. Levels of homocysteine are controlled mainly by diet, through the intake of methionine-rich and B-vitamin-family-rich (B6, folic acid and B12) food [2]. During aging, there is an increase in total plasma HCy levels within a range that is considered normal [3]. However, under age-associated pathological conditions, such as neurogenerative disease, there is an abnormal increase in the total HCy levels found in plasma and cerebrospinal fluid (CSF) [3,4]. Total plasma HCy concentrations in the range of 5–15 μM are considered to be normal, while 15–30 µM HCy is considered mild, and 30–100 µM is moderate. However, levels over 100 µM reflect severe homocysteinemia, a condition called hyperhomocysteinemia (HHCy) [5].

HHCy is a risk factor for vascular and neurodegenerative diseases [6,7,8,9] and was clinically followed in the Framingham Offspring Study for over 20 years [10]. An inverse correlation between total blood HCy and cognitive performance was found in patients over 60 years of age [10]. The two most common causes of dementia, in order of prevalence, are Alzheimer’s disease (AD) and vascular dementia (VaD) [11]. In a consensus statement, taking evidence from the last 20 years, it was concluded that the elevated total plasma levels of HCy cause dementia [12]. Dementia is a complex and multifactorial condition, but growing evidence points to HCy levels as relevant for the development of cognitive decline, dementia, and AD [10,12]. The early effects seen in AD patients include cerebral microangiopathy and endothelial dysfunction, particularly at the blood–brain barrier (BBB). Notably, it has been reported that HHCy causes increased BBB permeability [11,13,14], leading to increased CSF HCy levels, thus contributing to early defects in AD and dementia.

In 2015, we published a novel study demonstrating, for the first time, that an L-methionine rich diet, a known protocol to increase HCy levels [15], promotes the accumulation of Aβ species, inflammation, oxidative stress, and cognitive deficits in wild-type mice by increasing L-methionine in the diet [16]. Several mechanisms relating elevated HCy levels to the development of AD have been postulated, including: cerebrovascular pathology [17], oxidative stress [18,19,20], alterations in DNA methylation [21], endoplasmic reticulum stress [22], excitotoxic activation of NMDA receptors [23,24], increased tau phosphorylation [25], increased neurofibrillary tangles [17], and increased Aβ levels [21,26,27,28]. Unfortunately, most conclusions were achieved in different biological models using inconsistent HCy concentrations. Here, we study, for the first time, the effects of different HCy concentrations, in a consistent and exhaustive manner, over the CNS’s structure and function and its role in Aβ toxicity.

We performed studies to determine the effects of different HCy concentrations on oxidative stress, synaptic protein levels, and synaptic activity in hippocampal slices of two-month-old wild-type mice, as well as in vitro Aβ_1-40_ (Aβ_40_) aggregation kinetics. Our results show that: (1) High HCy concentrations cause an increase in oxidative stress, (2) HCy induces differential changes in the levels of pre- and post-synaptic proteins, (3) high HCy concentrations induce a toxic overactivation of excitatory transmission in the CA1 hippocampal region, and (4) low HCy concentrations cause slower Aβ_40_ aggregation, whereas elevated HCy increases the aggregation kinetics. Overall, these results indicate that HCy shows an adaptative response characterized by a biphasic dose response, which is consistent with a hormetic-like effect [29]. Here, we report that at low concentrations, HCy has beneficial effects, while at higher concentrations, it exerts neurotoxic effects that could be related to an AD-like pathology. Hormesis-like biphasic response patterns are commonly observed in biology but have not been commonly studied since, in most cases, research has focused on a specific concentration [30]. Our findings show that the hormetic-like response of HCy is fundamental to controlling preventable risk factors with high HCy levels. This underscores the relevance of maintaining a balance in HCy levels in order to achieve healthy aging or to prevent age-related pathologies, such as dementia and AD.

## 2. Results

### 2.1. Acute Treatment with High HCy Concentrations Increases Oxidative Stress

**Figure 1:**
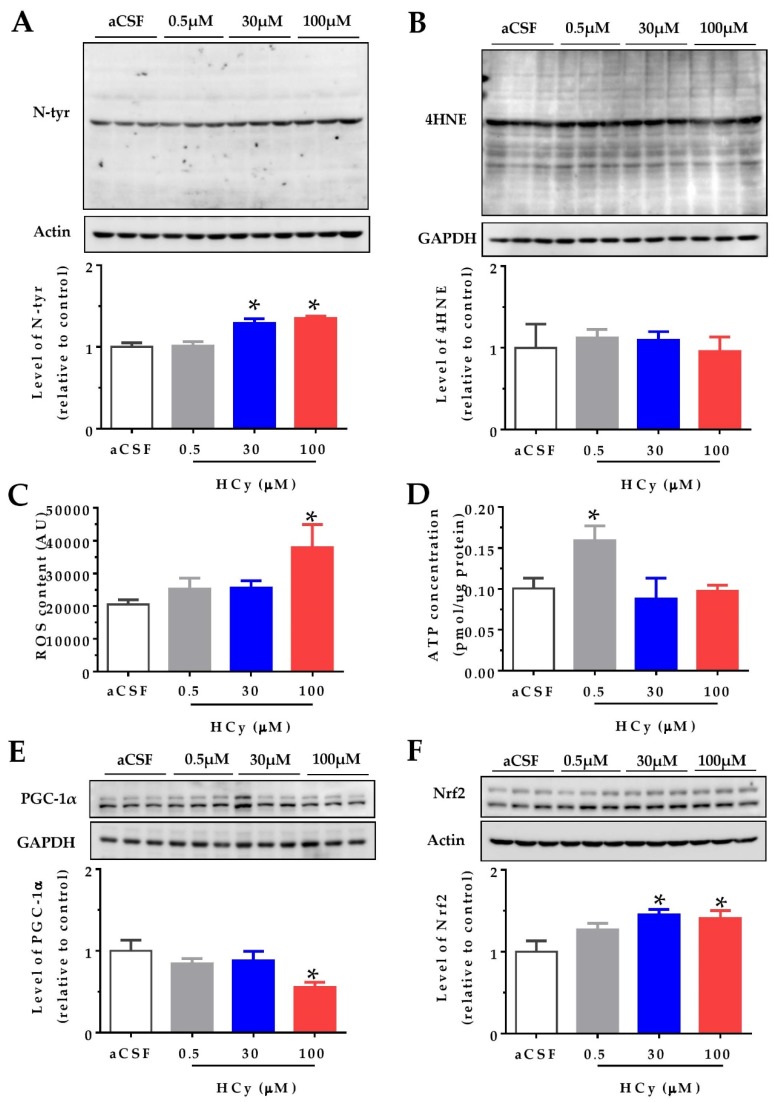
High concentrations of homocysteine (HCy) increase oxidative stress in the hippocampus. Levels of oxidative damage on hippocampal slices were evaluated after treatment with increasing concentrations of HCy (0.5, 30, and 100 µM) for 1 h. (**A**) Immunoblot against N-tyrosine (N-tyr) (above) and its densitometric analyses (below). (**B**) The 4-hydroxinonenal (4-HNE) antibody used to evaluate lipoprotein peroxidation levels (above) and their quantification (below). (**C**) Reactive Oxygen Species (ROS) content measured in the whole lysate using the fluorescent dye H2DCFDA. (**D**) ATP concentrations measured in the whole lysate using a bioluminescent ATP kit. (**E**) Levels of Peroxisome proliferator-activated receptor-gamma coactivator 1 alpha (PGC-1α) (above) and its quantification (below). (**F**) Immunoblot against nuclear factor erythroid 2-related factor 2 (Nrf2) (above) and quantification (below). Actin or GAPDH was used as the loading control. Protein levels were normalized against cerebrospinal fluid (CSF) solution-treated hippocampal slices. Each lane represents three independent samples (N = 3). A one-way analysis of variance (ANOVA) was performed followed by a Bonferroni post-test. Bars are the mean ± SEM. *p < 0.05.

It has been widely reported that HCy increases protein oxidation in the cerebral cortex and hippocampus (i.e., oxidative stress) in both in vitro and in vivo studies [1,6,20]. We studied the effects of oxidative stress indicators after acute HCy treatment (1 h) of hippocampal slices from two-month-old wild-type mice. Different doses of HCy (0.5, 30, and 100 µM in a CSF solution) were used, in order to study various physiological and pathological concentrations. Figure 1A shows that 30 and 100 µM HCy treatments caused an increase in the levels of N-tyrosine (N-tyr), a widely used marker for protein oxidation [31], in line with previous reports [16]. In contrast, no significant changes were detected using the antibody 4-hydroxinonenal (4-HNE), a marker for lipoprotein peroxidation, suggesting that HCy-induced oxidative damage is mediated by specific oxidant molecules [31]. To evaluate if oxidative damage is a consequence of increased levels of reactive oxygen species (ROS), we measured the ROS content in the whole lysate using the fluorescent dye H2DCFDA. A significant increase in ROS levels was detected after 1 h treatment with only 100 µM HCy (Figure 1C), proving that 100 µM HCy increases oxidative stress by increasing ROS production. Since mitochondria are the main ROS producer, increased ROS content could be a consequence of defective oxidative phosphorylation [32,33]. An increase in ROS content is related to mitochondrial metabolism and an increase in ATP levels. This remains true until excess stress in the mitochondria leads to the uncoupling of ATP and ROS levels, which is commonly seen in neurodegenerative diseases [33]. Therefore, we proceeded to determine ATP levels using a luminescent ATP kit. ATP levels were increased with 0.5 µM HCy, with no significant increase at 30 and 100 µM HCy (Figure 1D), suggesting that high HCy concentrations do not affect the bioenergetic metabolism. The transcriptional co-activator, peroxisome proliferator-activated receptor gamma coactivator 1-alpha (PGC-1α), regulates mitochondrial metabolism, antioxidant response, and energy function [34,35]. It has been described that PGC-1α levels are diminished in AD patients [36]. Accordingly, Figure 1E shows a decrease in PGC-1α levels at a 100 µM HCy concentration, with no effects seen at lower HCy concentrations. Another consequence of increased oxidative stress is the increment in protein levels and activation of the nuclear factor erythroid 2-related (Nfr2), which regulates the expression of antioxidant enzymes [37,38]. Figure 1F shows that at 30 and 100 µM, HCy increases Nfr2 levels, suggesting that Nrf2 is increased in response to oxidative stress and oxidative damage, as seen above. Overall, a 1 h treatment with 30 and 100 µM HCy promotes oxidative stress and modulates the oxidative response in mouse hippocampal slices by altering PGC1α and Nrf2 proteins.

### 2.2. Hormetic Effects of HCy on the Levels of Pre- and Post-Synaptic Proteins

**Figure 2:**
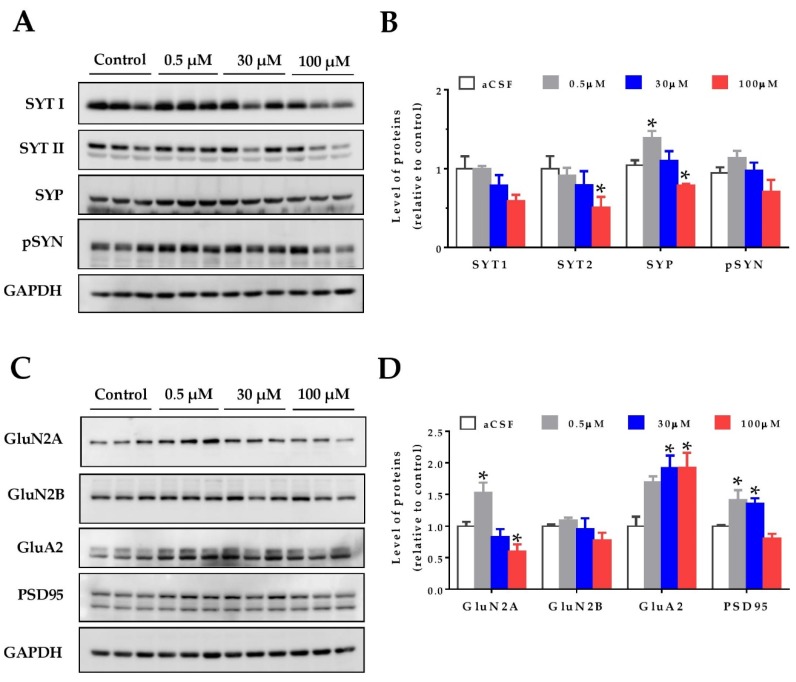
Homocysteine has a hormetic effect on synaptic protein levels of the hippocampus. The protein levels of hippocampal slices were evaluated after treatment with increasing concentrations of HCy (0.5, 30, and 100 µM) for 1 h. (**A**) The pre-synaptic proteins Synaptotagmin 1 (SYT1), Synaptotagmin 2 (SYT2), Synaptophysin (SYP), and phospho-Synapsin (p-SYN), were evaluated. (**B**) Densitometric analysis of (A) relative to the control condition. (**C**) Levels of post-synaptic protein NMDA receptor subunits 2A (GluN2A) and 2B (GluN2B), AMPA receptor subunit A2 (GluA2), and postsynaptic density 95 (PSD95) were evaluated. (**D**) A densitometric analysis of (C). In all cases, GAPDH was used as loading control, and protein levels were normalized against control CSF hippocampal slices. Each lane represents independent samples, n = 3. A one-way ANOVA was performed followed by a Bonferroni post-test. Bars are the mean ± SEM. * *p* < 0.05.

Oxidative stress can affect proteins and their cellular structures. For this reason, as a first approach to study the effects of homocysteine on the hippocampal synapses, we evaluated synaptic protein levels. Hippocampal slices from two-month-old wild-type mice were treated with increasing concentrations of HCy, and the protein levels were detected by immunoblot. As pre-synaptic markers, the levels of synaptotagmin 1 and synaptotagmin 2 (SYT1 and SYT2), Synaptophysin (SYP), and phospho-Synapsin (pS553-SYN) were evaluated (Figure 2A,B). All these proteins are part of the neurotransmitter release machinery [39]. The SYT11 levels showed no significant differences, whereas the levels of SYT2 were significantly decreased after 100 µM HCy treatment, with no significant changes at lower concentrations (Figure 2A,B). For the SYP levels, we observed a hormetic response, in which 0.5 µM HCy significantly increased SYP levels. Conversely, 100 µM HCy significantly decreased SYP levels (Figure 2A,B). The p-SYN levels showed a similar trend to SYP, without attaining significant differences. 

To analyze the post-synaptic compartment, the levels of the AMPA receptor subunit, GluA2, as well as the NMDA receptor subunits, GluN2A and GluN2B, and the postsynaptic density protein, PSD-95, were measured (Figure 2C,D). It has been widely noted that HCy targets the GluN2A subunits of NMDA receptors, acting as an agonist for these receptors and increasing the current amplitude [23,24,40,41]. Here, we see that at 0.5 µM HCy, GluN2A levels are increased, whereas at 100 µM HCy, there is a significant decrease, corroborating the dichotomous roles of HCy, depending on the concentration used (Figure 2C,D). GluN2B levels showed no significant changes, which is in line with the exclusive agonist action of HCy over GluN2A subunits (as previously reported), and not GluN2B subunits [24,42]. On the other hand, GluA2 subunit levels of the AMPA receptor were significantly increased at 30 and 100 µM HCy (Figure 2C,D). Even though GluA2 levels were not significantly different from those observed with a 0.5 µM treatment, we observed a linear correlation between HCy concentration and effect, unlike for the other proteins we observed. Also, after a 1 h treatment with 0.5 and 30 μM HCy, the levels of PSD-95 were significantly increased and were maintained at basal levels with 100 µM HCy. These results indicate that HCy has a differential effect on pre- and post-synaptic protein levels at low concentrations, increasing certain pre- and post-synaptic protein levels, whereas at high HCy concentrations, a decrease was observed, suggesting that HCy could modify the function of hippocampal neurons.

### 2.3. HCy Increases Field Excitatory Post-Synaptic Potentials (fEPSP) in the CA1 Hippocampal Region

**Figure 3:**
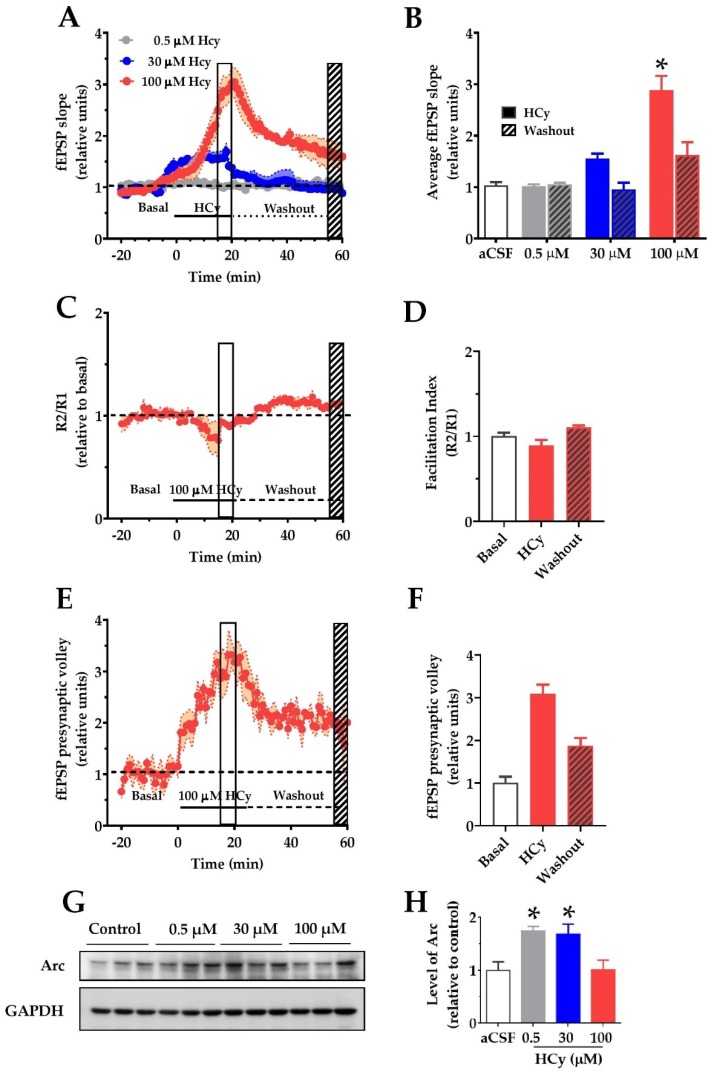
Homocysteine increases basal field excitatory post-synaptic potentials (fEPSP) amplitude in hippocampal slices. For recording of fEPSP amplitudes, HCy was bath-applied for 20 min in a bath (continuous line) at different concentrations followed by a 40 min washout on aCSF (dotted line). The open box marks the last 5 min of HCy treatment (15–20 min), and the scratched box marks the last 5 min of the washout (55–60 min). (**A**) Different concentrations of HCy over basal fEPSP (represented as a dotted line and quantified as the average fEPSP in (B)), (**B**) a plot of average fEPSP amplitude changes (relative to the aCSF control) according to (A). Only 100 µM HCy shows significant differences. After washout, 100 µM HCy does not completely return to basal levels. (**C**) A paired-pulse facilitation (R2/R1) test of 100 µM HCy. (**D**) Quantification of (C) shows no significant differences. (**E**) fEPSP of the pre-synaptic volley for 100 µM HCy treatment. (**F**) Quantification of (E) shows significant differences due to 100 HCy treatment. (**G**) Immunoblot of Arc, for different concentrations of HCy, with GAPDH as the loading control. (**H**) Densitometric analysis of (G), relative to the control condition. Bars are the mean ± SEM of a minimum of 8 different brain slices. * *p* < 0.05. A two-way ANOVA was performed followed by a Bonferroni post-test. Bars are the mean ± SEM. * *p* < 0.05.

To determine if changes in the synaptic structure result in functional alterations, we evaluated the electrophysiological response to different HCy concentrations. To do so, we recorded field excitatory post-synaptic potentials (fEPSP) over synaptic transmission in the CA3–CA1 circuitry in acute hippocampal slices. After 20 min of basal stable recording with aCSF, the perfusion was changed by different concentrations of HCy for 20 min (continuous line), after which the perfusion was changed back to aCSF (dotted line) to washout HCy (Figure 3A). The last 5 min of HCy treatment (empty box) and the last 5 min of washout (filled box) were averaged and plotted in Figure 3B. Only 100 µM HCy caused significant changes in the average fEPSP slope (Figure 3B). After washout with aCSF, this effect was not fully recovered to basal levels, which supports the idea that the effects of 100 µM HCy are, in fact, synaptotoxic. The magnitude of the response generated did not change the facilitation index (Figure 3C), a test used to evaluate presynaptic changes through a paired pulse stimulation protocol, indicating that the response is not dependent on an increase in presynaptic activity but is, instead, primarily mediated by postsynaptic components. Interestingly, we observed a significant change in the presynaptic volley slope (Figure 3E,F), which, together with Figure 3C, suggests that HCy may not affect neurotransmitter release. Instead, HCy may increase the number of active fibers recruited. To confirm these changes in activity, we analyzed the levels of the activity-regulated cytoskeleton-associated (Arc) protein, a postsynaptic protein and a member of the immediate-early genes (IEG) that are activated within minutes after firing activity [43]. For this reason, increased Arc levels are an indicator of neuronal activity. We observed a marked increase in Arc levels at 0.5 and 30 µM HCy and returns to basal levels with 100 µM HCy. This could be a sign of synaptotoxicity caused by a high HCy (100 µM) concentration, which agrees with the electrophysiology data showing high glutamatergic activity followed by an inability to return to basal levels after washout (Figure 3A,B).

### 2.4. Hormetic-Like Effects of HCy on the Aggregation Kinetics of Amyloid-β 1–40 Peptide

**Figure 4:**
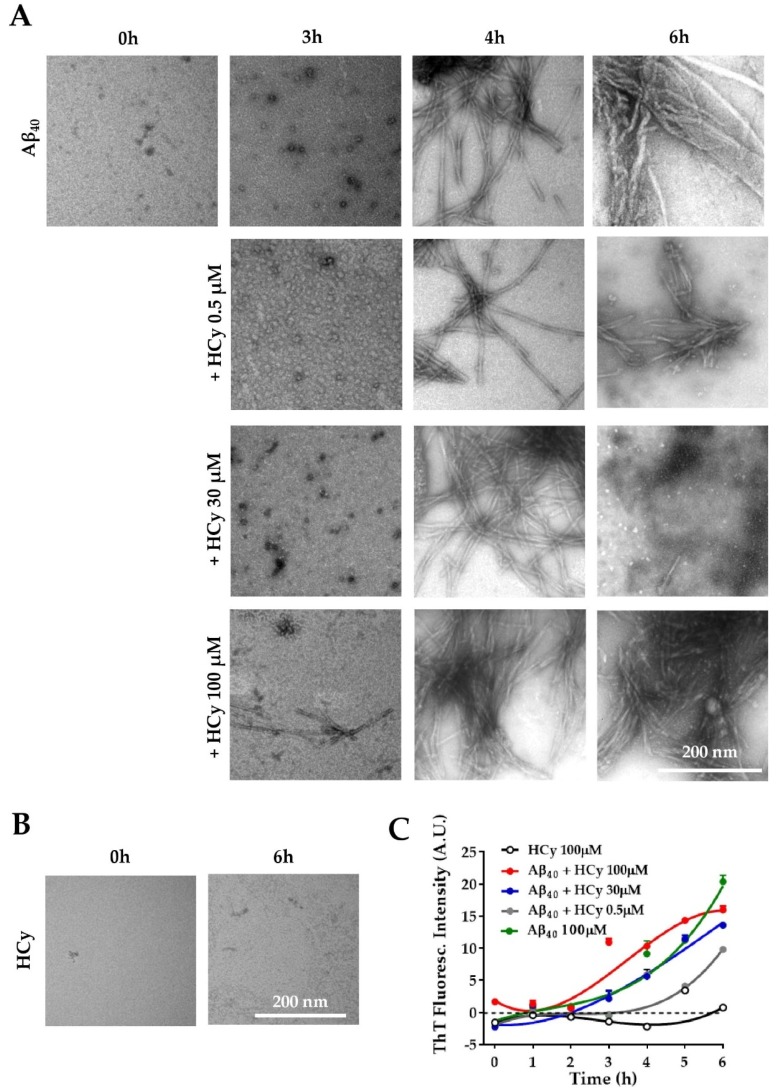
Differential effects of HCy on Aβ aggregation. (**A**) Electron microscopy of Aβ_40_ peptide added in the absence of HCy or after 3, 4, or 6 h of aggregation with different concentrations of HCy. (**B**) Representative electron microscopy images showing no aggregation of 100 μM HCy alone after 6 h. (**C**) The Aβ_40_ peptide aggregation curve followed by a Thioflavine-T (Th-T) fluorescence assay in the presence or absence of different concentrations of HCy. Curves were subjected to a robust fit using a third-order polynomial equation, with a goodness of fit RSDR of 1.11 for Aβ 100 µM, 0.66 for Aβ + HCy 0.5 µM, 1.89 for Aβ + HCy 30 µM, 1.60 for Aβ + 100 µM, and 0.56 for HCy 100 µM.

It has been reported that HHCy can cause an increase in Aβ load and cognitive deficits in vivo [16,44,45,46]. Ideas on the possible mechanism behind this phenomenon include enhanced expression of amyloidogenic γ-secretase [26] and increased expression of APP [26]. Here, we evaluated the possibility that HCy alters Aβ aggregation. To answer this question, the same concentrations of HCy used previously were incubated with 100 µM Aβ_40_ peptide at room temperature under constant stirring. To correlate the kinetic curve with specific types of aggregates, we used the Aβ_40_ peptide, which has been shown to present a more defined lag-phase, allowing us to recognize, through the kinetics, which Aβ species are present at the determined time points [47,48]. The aggregation kinetics of Aβ were followed for 6 h with the addition of a fluorescent dye, thioflavin-T (ThT), which detects the β-sheet structures of amyloid fibrils [49]. The aggregation states were observed by electron microscopy at different time points. Figure 4A shows representative electron microscopy images of the Aβ_40_ aggregation alone or co-incubated with HCy for 0, 3, 4, and 6 h. At the initial point of the aggregation course (0 h), Aβ_40_ alone was not aggregated and remained as monomers, which gradually formed oligomers (3 h), individual fibrils (4 h), and large aggregates (6 h), as expected [50]. Interestingly, higher concentrations of HCy accelerated the aggregation kinetics of Aβ_40_. For Aβ_40_ + 100 µM HCy, at 3 h, we observed the fast formation of aggregates, such as protofibrils and small fibrils, unlike Aβ_40_ alone. At 4 h of incubation, there was a dramatic increment in the presence of fibrils in comparison to those observed at the same time point for 0.5 µM HCy or Aβ_40_ alone. At 6 h, the Aβ_40_ + 100 µM HCy formed huge and dense aggregates. Most importantly, for the Aβ_40_ + 30 µM HCy condition, at the same time point, we observed small fibers and also oligomers, suggesting a disaggregation process. This could be very interesting and deserves to be further studied. The lowest HCy concentration clearly showed slower aggregation kinetics compared to every other condition, even when compared with Aβ_40_ aggregation alone. Moreover, the sizes of the Aβ_40_ fibrils formed in the presence of 0.5 µM HCy at 6 h were clearly smaller than those observed with Aβ_40_ alone, suggesting that this concentration of HCy delays Aβ_40_ aggregation. In Figure 4B, we controlled for the possibility that HCy may have formed aggregates by itself, obscuring the results. No HCy aggregates were observed after 6 h of aggregation (Figure 4B). Next, we followed the aggregation kinetics over time by measuring the fluorescence intensity of ThT. Figure 4C clearly shows that Aβ_40_ alone continuously aggregates. By comparison, the aggregation kinetics are faster under the Aβ_40_ + 100 µM HCy condition and slower under the Aβ_40_ + 0.5 µM HCy condition, as shown in Figure 4A, whereas the Aβ_40_ + 30 µM HCy kinetics present an intermediate rate of aggregation between the previous two conditions, similar to the aggregation kinetics observed for Aβ_40_ alone. This indicates that higher concentrations of HCy accelerate the aggregation process of Aβ_40_, forming larger aggregates. On the contrary, low concentrations of HCy seem to slow the aggregation kinetics, favoring the presence of smaller aggregates. This is in accordance with the conclusions of Figure 4A, suggesting that HCy has a differential effect on Aβ_40_ peptide aggregation, depending on the HCy concentration used. In this way, an increase in HCy levels, like the one seen in HHCy, would favor the formation of high molecular weight aggregates (i.e., fibrils), whereas low concentrations favor the formation of low molecular weight aggregates (i.e., oligomers). The present research is the first in vitro study on the direct effect of different HCy concentrations over Aβ_40_ aggregation. These groundbreaking results will help us understand the toxic mechanisms of HCy in amyloidogenic diseases.

### 2.5. A High Concentration of HCy Causes an Enhanced LTP Response and Increases Excitability after Aβ_42_-Induced Depression

**Figure 5:**
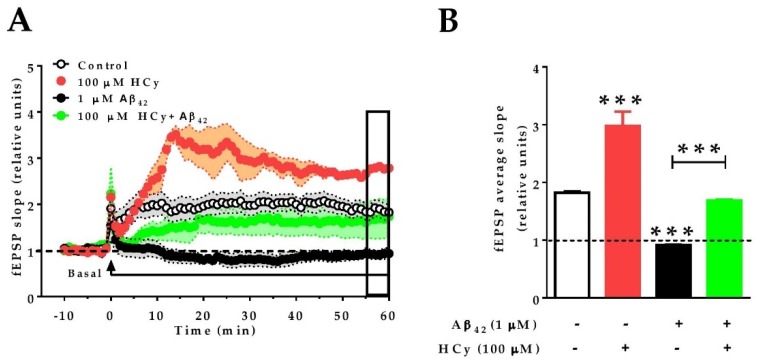
HCy increases the excitability of Aβ_42_-treated hippocampal slices. Recording of fEPSP amplitudes. (**A**) After 10 min of basal recording, long-term potentiation (LTP) was induced by theta burst stimulation (TBS) (arrow). At the same time, the correspondent treatment (control aCSF, 100 µM HCy, 1 µM Aβ_42_, or HCy + Aβ_42_) begins perfusion. Treatments were continuously administered, as shown by the horizontal line, for 60 min. The empty box at the end of the recording represents the last 5 min of data acquisition. (**B**) Quantification of the average fEPSP in the last 5 min of recordings. Bars are the mean ± SEM for three independent experiments with a minimum of eight different brain slices. A one-way ANOVA was performed followed by a Bonferroni post-test. Bars are the mean ± SEM. * *p* < 0.05, *** *p* < 0.001.

Finally, since 100 μM HCy showed the greatest differences in field potentiation and GluN2A levels, we evaluated whether this concentration of HCy can affect the long-term potentiation (LTP) of hippocampal slices subjected to theta burst stimulation (TBS). Similar to the effects under paired pulse stimulation (Figure 3A,B), 100 µM HCy enhanced LTP, with an increase in the amplitude (297.9% ± 0.24%) over the baseline, which was significantly higher than the control LTP (181.9% ± 0.02%) (Figure 5A,B). It has been previously described by our group and others that 1 µM Aβ_42_ oligomers completely abolish LTP [51,52]. Therefore, we evaluated the effect of HCy co-incubated with Aβ_42_ oligomers on LTP. We observed that Aβ_42_ generates an 8.3% ± 0.01% decrease of fEPSP compared to the baseline. Remarkably, when 1 μM Aβ_42_ oligomers plus 100 μM HCy are used, LTP is generated, with an intermediate effect of 168.7% ± 0.01% potentiation over the baseline—a similar value to the one observed under the control condition (181.9% ± 0.02%) (Figure 5A,B). Therefore, it is clear that 100 μM HCy is able to induce a strong increase in synaptic response over basal conditions. Since the data here suggest that 100 µM HCy is toxic (which agrees with the literature [40,53,54]), we propose that HCy + Aβ_42_ treatment is actually an effect of the toxic overactivation of glutamatergic activity rather than a protective effect of HCy over Aβ oligomers.

## 3. Discussion

In this study, we showed that HCy can exert protective or toxic effects depending on its concentration. We report that HCy at lower concentrations increases ATP production and synaptic protein levels while slowing Aβ_40_ aggregation. On the other hand, high HCy concentrations increase oxidative stress, decrease synaptic protein levels, and promote faster Aβ_40_ aggregation, among others. This non-linear dose-response behavior is called a hormetic effect. Hormetic-like effects of HCy were first described by Lipton et al. in 1997 [23]. Since then, over 20 years have passed and few articles have been published regarding this matter [40,54,55,56,57]. Unfortunately, most of the research done with HCy in the last two decades has used extremely high concentrations, disregarding the effects of lower HCy concentrations on synaptic functions. The rationale behind using high HCy concentrations is based on the total HCy plasma values, but not on the levels found in CSF. Under physiological conditions, average plasma HCy levels are 11.44 and 0.062 µM in CSF [3], a difference of three orders of magnitude. The same study found that, under pathological conditions, like in AD patients, plasma concentrations average 12.56 and 0.076 µM in CSF [3]. Different studies have also found diverse HCy levels for AD patients, in the range of 6.04–16.2 µM in plasma and 0.28–0.66 µM HCy in CSF [58]. This represents a problem, since the cutoff values are a matter of debate, and discrepancies exist within different publications.

Recently, high HCy levels were classified as a modifiable risk factor and a biomarker of AD and other types of dementia [12,59,60,61]. Given the importance of finding a modifiable risk factor like HCy, it is unfortunate that there are no consistent studies on the differential effects of HCy concentrations. Therefore, we studied different concentrations of HCy to further understand its role in physiological and pathophysiological conditions.

### 3.1. High HCy Concentrations Induce Oxidative Stress and Alterations in Mitochondrial Metabolism

We detected increased n-Tyr levels with 30 and 100 µM HCy, as well as increased ROS content in hippocampal slices (Figure 1). Intriguingly, we detected significant changes in N-tyr but not at 4-HNE levels. One possible explanation for this result is that the action mechanism of HCy affects preferentially protein nitro-tyrosilation but not lipid peroxidation. Alternatively, it is possible that protein oxidation, detected by N-tyr, is either more sensitive to HCy or occurs more quickly than lipid oxidation, as detected by 4-HNE.

An increase in Nrf2 levels, an antioxidant response protein, corroborates that 1 h exposure to high HCy concentrations (30–100 µM) is enough to induce an increase in oxidative stress. Since we are examining the response in whole hippocampal slices with diverse cell types, we cannot pinpoint which cell type is responsible for the elevated Nrf2 levels. Nonetheless, it has been described that retinal Müller glial cells, and not retinal ganglion cells, are the cells responsive to oxidative stress, with an increase in Nrf2 levels [20]. Therefore, it is possible that glial cells, rather than neurons, in these hippocampal preparations were responsible for the increase in Nrf2 levels. We also found that high HCy concentrations decrease PGC1α levels. Interestingly, PGC1α controls mitochondrial biogenesis, and its expression increases in response to oxidative stress [62]. Therefore, it is possible that by causing a decrease in PGC1α levels, HCy increases ROS levels, leading to exacerbated oxidative stress.

### 3.2. HCy Follows a Hormetic-Like Effect and Differentially Affects Synaptic Protein Levels

We found that exposure to 0.5 μM HCy generates an increase in the levels of PSD95 and its subunits GluN2A and GluA2. Higher concentrations of HCy seem to cause a decrease in the relative levels of GluN2A and the pre-synaptic proteins SYT1 and SYP, and similar effects were noted by Chai et al. [63]. HCy has opposite effects depending on whether it is found at low or high concentrations, a phenomenon called hormesis. Low concentrations of HCy act as a partial antagonist of the glycine site in NMDA receptors, whereas high concentrations of HCy act as an agonist of the glutamate binding site in NMDA receptors [23]. Interestingly, antagonists of the glycine binding site have been described to have therapeutic uses for pain, stroke, and dementia, among other functions [64]. Therefore, by acting as a partial antagonist of the glycine binding site, low doses of HCy could exert protective and completely opposite effects to higher HCy concentrations. This is consistent with our observations of increased SYP, GluN2A, and PSD95 levels after exposure to 0.5 µM Hcy (Figure 2). On the contrary, high HCy concentrations (100 µM) significantly reduced the protein levels of SYT2, SYP, and GluN2A. Indeed, GluN2A levels seem to be particularly sensitive to HCy levels, which again recalls the aforementioned interaction of NMDA receptors with HCy [23,40,65].

### 3.3. The HCy-Induced Increase in fEPSP Might be Related to NMDA-Dependent over-Activation and Consequent Excitotoxicity

In hippocampal slices of wild-type mice, we observed that high levels of HCy increase the fEPSP. A concentration-dependent effect is induced by HCy, in which lower concentrations (i.e., 0.5 to 30 µM) have a gradient effect on fEPSP. There is an important body of evidence that demonstrates HCy to cause an increase in NMDA currents, which could lead to excitotoxicity [23,24,65,66]. Moreover, since slices treated with 100 µM HCy were unable to return to basal levels after washout, we believe that 100 µM HCy exerts excitotoxic effects related to NMDAR overactivation. It has been reported that HCy can bind to NMDA receptors due to its homology with the ligand NMDA [67]. This is normally followed by a fast activation of the receptors leading to increased intracellular calcium concentrations. In this way, a plethora of mechanisms can be triggered. Among these mechanisms (which must be further explored) are CaMKII activation, calcineurin activation, and calcium-induced calcium release by internal storages, among many others. Therefore, HCy exerts dual effects depending on its concentration. At low concentrations, HCy acts as a partial antagonist of the glycine site of NMDARs, and, at high concentrations, it acts as an agonist of the glutamate site of NMDARs [23,40].

### 3.4. HCy Affects Aβ_40_ Aggregation Kinetics and Toxicity

High HCy levels have long been a proposed risk factor for AD and dementia [12,68,69]. The neurotoxicity of the Aβ peptide is dependent on its conformation, quaternary structure, and the morphology of the bundles formed during the aggregation process [70,71]. Aβ_42_ is the most neurotoxic species of Aβ peptide found in patients with AD [72]. However, when studying the in vitro aggregation kinetics of Aβ_o_*,* it is often preferable to use the Aβ_40_ peptide [73,74,75] because Aβ_42_ has much faster aggregation kinetics than Aβ_40_ [47,48]. Aβ_40_ also has a longer lag phase that allows for the clear identification of monomeric, oligomeric, and fibrillar forms, which would not be clearly identifiable when using Aβ_42_ [47,48]. For this reason, we performed our aggregation studies on Aβ_40_ rather than Aβ_42_ (Figure 4). To the best of our knowledge, there is only one work studying the effects of HCy over Aβ aggregation. This study found that high HCy concentrations induced the formation of β-sheet structures in Aβ and consequently favored β-fibrils [76], which agrees with our results. We also found that a low HCy concentration (0.5 μM) inhibits the aggregation of Aβ_40_ oligomers, reflecting a neuroprotective effect of low HCy concentrations. Higher concentrations of HCy (30 and 100 µM), co-incubated with the same amount of Aβ_40_ oligomers, clearly produced larger aggregates (Figure 4).

On the another hand, it has been established that Aβ_42_ dimmers, trimers, tetramers, or high molecular weight oligomers are more toxic to neurons, than monomeric species [77,78]. Therefore, HHCy would contribute to the Aβ_42_-mediated neurotoxicity. We also sought to test the functional effect of HCy and Aβ_42_. We found that when Aβ_42_ + 100 µM HCy were perfused to hippocampal slices, the LTP response was similar to that found at basal levels (Figure 5), unlike what happens with Aβ_42_ or HCy alone. Nonetheless, it is worth noting that the early phase of LTP shows different behavior compared to the control, finally reaching similar levels in late-phase LTP. This is not surprising considering the two very different molecular mechanisms involved in early LTP and late LTP. This intermediate effect of the potentiating action of HCy and the depressing action of Aβ_42_ could have several explanations. We propose that Aβ_42_ and HCy form a complex that accelerates the aggregation kinetics of Aβ_42_, favoring the formation of larger aggregates, as seen for Aβ_40_ (Figure 4A). Functionally, these aggregates cause an initial fEPSP decrease in early LTP followed by a more classical late LTP response comparable to the control levels. The exact molecular mechanism involved in this response remains unclear, but, in general, early LTP is related to CaMKII and rapid receptor trafficking [79]. This should be further studied to determine the effect of the Aβ_42_–HCy complex on LTP. In parallel, the formation of the Aβ_42_–HCy complex decreases the concentration of freely available HCy, which would also explain why we observed a lesser effect with fESPSP (compared to HCy alone), which avoids the HCy-induced excitotoxicity caused by high HCy concentrations. This would explain why we do not observe the depression caused by Aβ_42_ alone nor the glutamatergic overactivation (i.e., increased fEPSP) caused by HCy alone.

### 3.5. Final Conclusions and Perspectives

Altogether, we have established that low concentrations of HCy (1) increase ATP production, (2) increase synaptic protein levels, and (3) slow Aβ_40_ aggregation, whereas high concentrations of HCy (1) cause an increase in oxidative stress, (2) reduce the levels of pre-synaptic and post-synaptic proteins, (3) increase fEPSP over the basal condition and LTP (likely related to NMDAR overactivity), and (4) favor Aβ_40_ aggregation, a condition known to be toxic. Altogether, our results show the hormetic-like effects of HCy over neuronal structure and function, which are perceived to have beneficial properties at low concentrations and toxic effects at high concentrations. From this data, we cannot determine the exact threshold concentration at which HCy stops being beneficial and begins to be toxic. Thus, a more detailed analysis should be performed.

Monitoring HCy levels and treating HHCy should become the gold standard procedures for the elderly population. This would not only make it easier to detect at-risk populations but would also allow medical professionals to create personalized therapies according to the needs of each patient. This treatment, an increase in the intake of B family vitamins, would also provide an easy and affordable way to tackle dementia [12].

## 4. Materials and Methods

### 4.1. Antibodies

The primary antibodies used were anti-3-nitrotyrosine (A21285, Invitrogen, Waltham, USA), goat anti-4HNE (H6275-02, US Biological, Life Sciences, Salem, USA), mouse anti-Nrf2 (A-10) (sc-365949, Santa Cruz Biotechnology, Dallas, USA), mouse anti-PGC1α (4A8; sc-517380, Santa Cruz Biotechnology), mouse anti-Syt1/2 (H-9) (sc-393392, Santa Cruz Biotechnology), mouse anti-Syp (D-4; sc-17750, Santa Cruz Biotechnology), goat anti-pSyn Ia/b (Ser 553; sc-12913, Santa Cruz Biotechnology), mouse monoclonal anti-PSD95 (clon K28/43, UC Davis/NIH NeuroMab Facility, Davis, USA), mouse anti-GluN2A (E-4, sc-515148, Santa Cruz Biotechnology), mouse anti-GluN2B (A-8; sc-365597, Santa Cruz Biotechnology), mouse anti-GluA2 (clone L21/32; UC Davis/NIH NeuroMab Facility), goat anti-synaptophysin (sc-7568, Santa Cruz Biotechnology), and rabbit polyclonal anti-Arc (156 003, Synaptic Systems, Goettingen, Germany). Anti-Actin (sc-47778, Santa Cruz Biotechnology) or GAPDH (sc-25778, Santa Cruz Biotechnology) were used as the loading control.

### 4.2. Peptides and Reagents

Synthetic Aβ_1-40_ and Aβ_1-42_ peptides, corresponding to human sequences, were obtained from Genemed Synthesis, Inc. (San Francisco, CA, USA). Aβ peptide stock solutions were prepared by dissolving freeze-dried aliquots of Aβ in 1,1,1,3,3,3-hexafluoro-2-propanol (HFIP) at 1 mM. Immediately before use, the peptide film was dissolved in dimethyl sulfoxide (DMSO) at 5 mM and was then diluted in PBS to a final concentration of 100 μM [80]. Thioflavin-T, L-Homocysteine (69453), HFIP, and DMSO were obtained from Sigma Chemical Co. (St Louis, MO, USA). L-Homocysteine was dissolved in distilled water to a stock concentration of 350 mM and stored at −20 °C.

### 4.3. Immunoblotting

Brain slices treated with HCy or control were dissected on ice and immediately frozen at −150 °C or processed, as detailed previously. Briefly, the slices were homogenized in a RIPA buffer (50 mM Tris-Cl, pH 7.5, 150 mM NaCl, 1% NP-_40_, 0.5% sodium deoxycholate, and 1% SDS) supplemented with a protease inhibitor cocktail (Sigma-Aldrich P83_40_) and phosphatase inhibitors (50 mM NaF, 1 mM Na3VO4 and 30 μM Na4P207) using a Potter homogenizer and then passed sequentially through different caliber syringes. Protein samples were centrifuged at 14,000 rpm at 4 °C twice for 15 min. Protein concentrations were determined using a BCA Protein Assay Kit (Pierce Biotechnology, Rockford, IL, USA). Twenty micrograms of samples were resolved by 10% SDS-PAGE and transferred to a PVDF membrane. These reactions were followed by incubation with anti-mouse, anti-goat, or anti-rabbit IgG peroxidase-conjugated antibodies (Pierce, Rockford, IL, USA) developed using an ECL kit (Western Lightning Plus ECL, PerkinElmer).

### 4.4. Measurement of ATP Concentration

ATP concentration was measured in hippocampal tissue lysates obtained with a Triton buffer (5 mM Tris, 150 mM NaCl, 1 mM EDTA, 1% (v/v) Triton X-100, pH = 7.4) using a luciferin/luciferase bioluminescence assay kit (ATP determination kit no. A22066, Molecular Probes, Invitrogen) [81,82,83]. The amount of ATP in each sample was calculated from the standard curves and normalized based on the total protein concentration.

### 4.5. Measurement of ROS Content

ROS content was measured using the fluorescent dye CM-H2DCFDA. Briefly, hippocampal samples diluted in Triton Buffer were added to a black 96-well plate in duplicate followed by the addition of 25 μM DCF. Then, the plate was incubated for 5 min and examined in a BioTek Synergy HT.

### 4.6. Thioflavin-T-Based Fluorometric Assay

Aliquots of Aβ_1_-_40_ peptide at the indicated concentrations were diluted in PBS pH 7.4 and incubated at different times under constant stirring (1200 rpm) at room temperature. Aβ_1-40_ was used to measure the kinetics since it has a slower aggregation curve, which allows for the identification of different aggregation states. For co-incubation experiments, homocysteine (HCy) was used at 0.5, 30, and 100 μM. To quantify the amyloid formation, the Th-T fluorescence method was used [84]. Briefly, following the incubation of Aβ_1-40_ alone or in the presence of HCy, the samples were taken every hour and diluted in 50 mM sodium phosphate buffer pH 6.0 and 0.1 mM Th-T. The fluorescence was monitored at an excitation of 450 nm and an emission of 485 nm using a JASCO spectrofluorometer. The binding of Th-T to amyloids produces a shift in its emission spectrum and an increase in the fluorescence signal, which is proportional to the number of amyloid fibrils formed.

### 4.7. Preparation of Aβ Oligomers

Synthetic Aβ_42_ peptides corresponding to wild-type human Aβ were obtained from Genemed Synthesis, Inc. (San Francisco, CA). An Aβ peptide stock solution was prepared by dissolving freeze-dried aliquots of Aβ in 1,1,1,3,3,3-hexafluoro-2-propanol (HFIP, Sigma H-8508) at 1 mM. For oligomer preparation, the peptide film was dissolved in dimethyl sulfoxide (DMSO, Sigma D2650) at 5 mM and was then diluted in PBS to a final concentration of 100 μM. This preparation was incubated overnight to allow the formation of Aβ oligomers and then centrifuged (14,000 rpm, 1 h, 4 °C) to eliminate any formed fibril [85]. An aliquot of the oligomer solution was used to quantify protein with a Qubit 2.0 Fluorometer (Invitrogen, Carlsbad, CA, USA) to determine the final concentration (70–80 μM). All new batches of Aβs were tested for quality control through electron microscopy and Tris-Tricine SDS gel electrophoresis, as previously described [80].

### 4.8. Slice Preparation and Electrophysiology

Hippocampal slices were prepared according to standard procedures, from 2-month-old male mice. Transverse slices (350 µm) from the dorsal hippocampus were cut under freshly prepared cold artificial cerebrospinal fluid (ACSF, in mM: 124 NaCl, 2.6 NaHCO_3_, 10 D-glucose, 2.69 KCl, 1.25 KH_2_PO_4_ 2.5 CaCl_2_, 1.3 MgSO_4_, y 2.60 NaHPO_4_) using a vibratome (Leica VT 1000s, Germany) and incubated in ACSF for 1 h at room temperature. From dissection onwards, the slices were constantly maintained under 5% CO_2_/95% O_2_ conditions. In all experiments, picrotoxin (PTX; 10 µM) was added to the ACSF perfusion media to suppress inhibitory GABA_A_ transmission. Slices were then transferred to an experimental chamber (2 mL), superfused (3 mL/min, at 22–26 °C) with gassed ACSF, and visualized by trans-illumination with a binocular stereomicroscope (MSZ-10, Nikon, Melville, NY). The experiments were carried out at room temperature (21–22 °C), measured at the recording chamber. To evoke field excitatory postsynaptic potentials (fEPSPs), SC fibers were activated by bipolar cathodic stimulation generated by a stimulator electrode (Axon 700b, Molecular Devices, Sunnyvale, CA) and connected to an isolation unit (Isoflex, AMPI, Jerusalem, Israel) [86]. Bipolar concentric electrodes (Platinum/Iridium, 125 µm OD diameter, FHC Inc., Bowdoin, ME) were placed in the stratum radiatum 100–200 µm from the recording site. The paired pulse facilitation index was calculated by ((R2-R1)/R1), where R1 and R2 were the peak amplitudes of the first and second fEPSP, respectively [51]. To generate LTP, we used a theta burst stimulation (TBS) consisting of 5 trains of stimulus with an inter-train interval of 20 s. Each train consisted of 10 bursts at 5 Hz, each burst having 4 pulses at 100 Hz. Recordings were filtered at 2.0–3.0 kHz, sampled at 4.0 kHz using an A/D converter, and stored with pClamp10 (Molecular Devices). Evoked postsynaptic responses were analyzed off-line using the pClampfit analysis software (Molecular Devices), which allowed visual detection of the events (computing only the events that exceeded an arbitrary threshold).

### 4.9. Electron Microscopy

Electron microscopy was performed as previously described [50]. Fresh aliquots of the samples were diluted 1:3 in water, and 5 μL were placed on Formvar/Carbon coated 300-mesh copper grids (1753-F, Ted Pella Inc, CA, USA) for 1 min. Excess amounts of the sample were removed with a drop of water for 3 min, and 5 μL of 2% aqueous uranyl acetate was placed onto the grid for 2 min, followed by the removal of excess staining solution with filter paper and air-drying. Observations were carried out using Philips Tecnai 12 electron microscope operated at 80 kV from the Microscopy Facility Unit of the Pontificia Universidad Católica de Chile. Photographs were taken at the original magnification of 60,000×.

### 4.10. Statistical Analysis

Data analysis was performed using the Prism 8 software (GraphPad Software Inc.) All results are expressed as the mean ± standard error. Data were tested for normality using Shapiro–Wilkinson and Kolmogorov–Smirnov tests. For statistical analysis, normally distributed data were analyzed by a one-way ANOVA with post hoc tests performed using the Bonferroni test. Non-normally distributed data were analyzed by a Kruskal–Wallis test with post hoc tests performed using Dunn’s test.
